# Serous Cavity Mast Cells Depend on the ROQUIN Paralogs

**DOI:** 10.1002/eji.70110

**Published:** 2025-12-19

**Authors:** Klaus Heger, Ali Masjedi, Assa Yeroslaviz, Theodor Zeng, Seren Baygün, Angela Vicente‐Luque, Chia‐I. Lien, Lena Osswald, Dieter Saur, Daniel Kovacs, Marc Schmidt‐Supprian

**Affiliations:** ^1^ Institute of Experimental Hematology School of Medicine and Health Technical University of Munich Munich Germany; ^2^ Max‐Planck Institute of Biochemistry Planegg Germany; ^3^ Present address: Genentech South San Francisco California USA; ^4^ Center For Translational Cancer Research (TranslaTUM) School of Medicine and Health Technical University of Munich Munich Germany; ^5^ Department of Medicine III School of Medicine and Health Technical University of Munich Munich Germany; ^6^ Chair of Translational Cancer Research and Institute of Experimental Cancer Therapy School of Medicine and Health Technical University of Munich Munich Germany

**Keywords:** connective tissue mast cells, degranulation, posttranscriptional gene regulation, pro‐inflammatory cytokines, ROQUIN paralogs, serous cavities

## Abstract

Mast cells are evolutionarily ancient immune cells located at strategic entry points for pathogens and allergens. Allergen exposure activates signal transduction pathways resembling those downstream of antigen receptors in T and B lymphocytes, leading to mast cell degranulation and cytokine secretion. The paralogous RNA‐binding proteins ROQUIN‐1 and ROQUIN‐2 prevent aberrant T cell activation and differentiation and are cleaved upon antigen receptor engagement. Here, we investigated their roles in connective tissue mast cells using conditional gene knockout in mice. We show that ROQUIN‐1 and ROQUIN‐2 are dispensable for skin mast cell development and maintenance, while they are essential for serosal mast cells residing in the peritoneal and pleural cavities. Concurrent ablation of both paralogs did not affect mast cell degranulation in vitro and in vivo, nor did it alter activation‐induced secretion of TNF and IL‐6, cytokines that are regulated by ROQUIN proteins in other cell types. Furthermore, we globally define ROQUIN‐regulated mRNAs in mast cells, and validate *Runx1t1* and *Ebi3* as indirect and *Lfng* as direct ROQUIN targets. Collectively, our results highlight the essential function of ROQUIN in connective tissue mast cells in serosal cavities.

## Graphical abstract

The RNA‐binding ROQUIN paralogs are critical immune regulators. We demonstrate that mast cell‐specific deficiency for ROQUIN‐1 and ROQUIN‐2 in mice leads to loss of mast cells in serous cavities. ROQUIN‐1 and 2 ablation does not affect mast cell degranulation or proinflammatory cytokine secretion, although they regulate many mRNAs directly or indirectly.

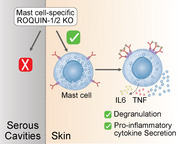

**Abbreviations**
BM: bone marrow
cPMC: cultured (mouse) peritoneal mast cells
DNP: 2,4‐dinitrophenyl
FCERIA: Fcε receptor Iα
HSA: human serum albumin
IgE: immunoglobulin E
MACS: magnetic activated cell sorting

## 1 Introduction

Mast cells (MCs) are long‐lived, tissue‐resident immune cells strategically positioned at host–environment interfaces, including the skin, mucosal surfaces, and serous cavities [1, 2]. They play a crucial role in detecting and responding to external as well as endogenous stimuli, including allergens, cytokines, neuropeptides, alarmins, and pathogen‐associated molecular patterns [1]. However, in atopic individuals, MC can drive allergic reactions by releasing a large array of mediators via a process termed degranulation, leading to itching, swelling, and in severe cases, anaphylaxis [2]. MCs capture circulating immunoglobulin E (IgE) via binding to their high‐affinity Fcε receptor Iα chain (FCERIA), and allergic triggering of this acquired antigen receptor module induces signaling similar to B and T cell receptors, culminating in degranulation and cytokine secretion [3]. Furthermore, MCs can contribute to chronic inflammation in conditions such as asthma and atopic dermatitis [2]. In rodents, MCs are roughly subdivided into connective tissue MCs (CTMCs) and mucosal MCs (MMCs) based on their anatomical location and specific MC protease content [4]. The highly granular CTMCs, rich in histamine and heparin, are abundant in the skin, serous cavities, and gastrointestinal submucosa. In contrast, MMCs are smaller and less granular and are predominantly found in the gut and respiratory epithelium [4]. Although all CTMCs share a core MC signature, MCs from different tissues exhibit distinct transcriptional profiles [5]. Notably, skin MCs and peritoneal MCs show significant transcriptional differences, including the expression of metalloproteinases, cell surface receptors, cytokines, and transcription factors [5].

While major transcriptional mechanisms governing MC development have been identified [6, 7, 8], the role of posttranscriptional gene regulation remains poorly understood. RNA‑binding proteins (RBPs) orchestrate post‑transcriptional control, allowing for rapid cellular adaptations to dynamic inflammatory contexts [9]. A recent study revealed that the RBPs REGNASE‐1 and REGNASE‐3 modulate MC survival and inflammatory responses [10]. ROQUIN‐1 and 2 (encoded by *Rc3h1* and *2*) are key paralogous RBPs that repress mRNA stability and translation in immune cells and other cell types, often in collaboration with REGNASE proteins [11], [12]. Although ROQUIN proteins share target mRNAs, they also exert nonredundant functions [11, 12]. ROQUIN proteins recognize conserved stem‐loop structures commonly within the 3'‐untranslated regions (3'‐UTRs) of target mRNAs [13]. Mutations in these stem‐loops abrogate ROQUIN‐mediated repression of the respective target mRNA, leading to enhanced protein expression [12, 13].

The ROQUIN paralogs bind to many mRNAs and are especially known to suppress immunologically relevant mRNAs, including transcription factors, cell surface receptors, signaling proteins, and cytokines [12, 13, 14, 15, 16], many of which are also expressed in MCs. Furthermore, *Adamts1* mRNA, a metalloprotease exclusively expressed in skin mast cells [5], binds to ROQUIN‐1 [13]. In T and B cells, antigen receptor engagement induces the cleavage of ROQUIN proteins by the paracaspase MALT1, thereby relieving target mRNA repression [17]. In MCs, antigenic activation of the IgE:FCER complex via the scaffolding function of MALT1 leads to production of TNF and IL‐6 [18], both of whose mRNAs are also repressed by ROQUINs [13, 16].

Employing conditional gene targeting in mice, we demonstrate that ROQUIN proteins are redundantly required for the presence of serosal MCs. We also reveal that ROQUIN proteins are dispensable for MC degranulation in vitro and in vivo, and while they do not significantly impact activation‐induced inflammatory cytokine secretion, they significantly shape posttranscriptional gene regulation in MCs.

## 2 Results and Discussion

### 2.1 Mast Cell‐Specific Ablation of ROQUIN‐1 and 2 Leads to Loss of Mature Mast Cells in Serosal Cavities

To investigate the role of the ROQUIN paralogs in MC biology, we generated MC‐specific ROQUIN‐1 knockout (KO) (Mcpt5‐Cre Rc3h1^F/F^), ROQUIN‐2 KO (Mcpt5‐Cre Rc3h2^F/F^), and double‐knockout (dKO) (Mcpt5‐Cre Rc3h1^F/F^ Rc3h2^F/F^) mice. Mcpt5‐Cre transgenic mice served as controls, as previous analyses showed no significant differences from wild‐type (WT) mice [19].

**FIGURE 1:**
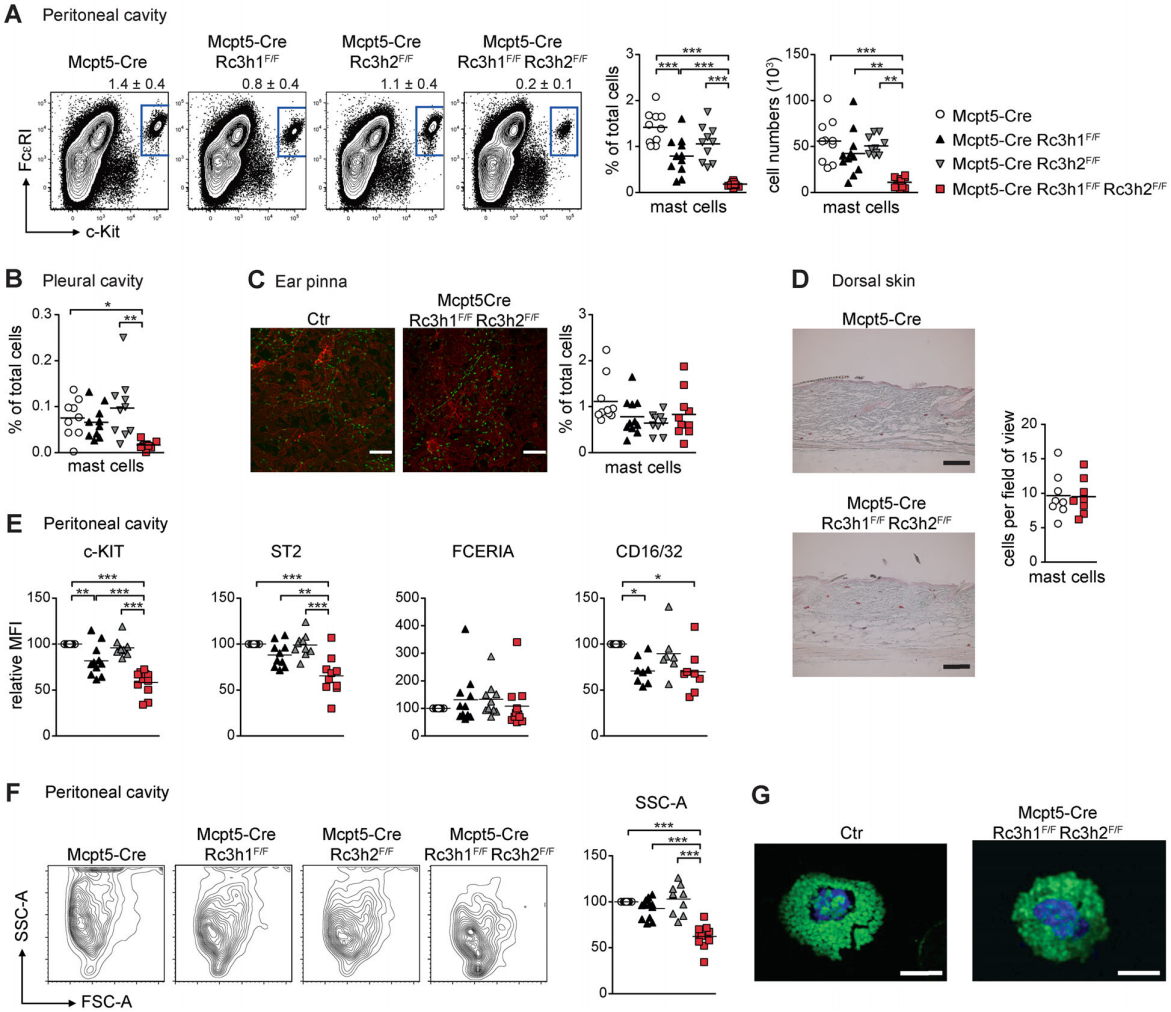
MC‐specific ROQUIN‐1/2‐deficiency reduces MC numbers and maturity in serous cavities (A) Representative flow cytometry plots of MCs in the peritoneal cavity of control (Mcpt5‐Cre), MC‐specific ROQUIN‐1 knockout (Mcpt5‐Cre Rc3h1^F/F^), ROQUIN‐2 knockout (Mcpt5‐Cre Rc3h2^F/F^), and ROQUIN‐1 and 2 double knockout (Mcpt5‐Cre Rc3h1^F/F^ Rc3h2^F/F^) mice. Numbers above the plots represent means ± SD. Scatter plots show MC percentages and absolute numbers; bars represent means. Data are from at least 10 mice per genotype. (B) Scatter plot depicting the percentages of MCs in the pleural cavity from at least 10 mice per genotype. Bars represent means. (C) Representative (of seven mice per genotype) immunofluorescence images of whole‐mount ear explants (avidin‐FITC, red: anti‐laminin; scale bar = 200 µm). The scatter plot shows the percentage of MCs (CD45^+^, FCERIA^+^, c‐Kit^+^) in ear skin digests, quantified by flow cytometry. (D) Representative histological sections of dorsal skin (scale bar = 100 µm) stained with alcian blue and safranin red and a corresponding scatter plot displaying MC frequencies. Each data point represents the mean MC count in 10 fields of view per mouse. Bars indicate means from at least eight mice per genotype. (E) Scatter plots displaying the mean fluorescence intensity (MFI) of MC markers normalized to MCs from Mcpt5‐Cre control mice. Bars indicate means from at least eight mice per genotype. (F) Representative flow cytometry plots showing side scatter (SSC‐A corresponds to MC granularity) versus forward scatter (FSC‐A corresponds to MC size) in the peritoneal cavity. The scatter plot shows the SSC‐A values of MC normalized to Mcpt5‐Cre MCs across genotypes. Bars indicate means from at least eight mice per genotype. (G) Representative immunofluorescence images (three mice per genotype) of FACS‐purified primary peritoneal MC (PMCs) (green: avidin‐FITC, blue: DAPI) (scale bar = 10 µm). Statistical significance: *p < 0.05, **p < 0.01, ***p < 0.001 (Student's *t*‐test using Welch correction or one‐way ANOVA).

Initially, we assessed the MC populations in the peritoneal and pleural cavities by flow cytometry and cytospins. ROQUIN‐1 deficiency led to a slight MC reduction in the peritoneal cavity, while ROQUIN‐2 knockout showed no effect (Figure 1A, Figure S1A). In contrast, MC‐specific ROQUIN‐1/2 dKO mice showed a three‐ to fourfold decrease in both relative and absolute MC numbers in peritoneal and pleural cavities (Figure 1B; Figure S1A,B). To evaluate skin‑resident MCs, we performed immunofluorescence microscopy on whole‑mount ear explants (Figure 1C) and histochemical analyses of dorsal skin sections (Figure 1D; Figure S1C). Neither ROQUIN single KOs nor dKO altered skin MC numbers or localization. Quantitative real‐time PCR and crosses to the coxsackie adenovirus receptor (CAR)‐based Cre reporter mouse strain [20] confirmed efficient Mcpt5‐Cre‐mediated recombination of conditional *Rc3h‐1/2* alleles (Figure S1D,E). Flow cytometric phenotyping revealed that ROQUIN‐1/2 dKO peritoneal MCs display an immature phenotype, with significant reductions in c‐KIT (CD117), ST2, and CD16/32 surface expression (Figure 1E) as well as reduced granularity assessed by side scatter (Figure 1F; Figure S1F) and avidin‐FITC staining of MC granules (Figure 1G). ROQUIN‐1‐deficient MCs also exhibited reduced expression of c‐KIT and CD16/32. Similar alterations were observed in pleural cavity MCs (Figure S1G). In contrast, primary skin MCs from ROQUIN single KOs and dKO displayed normal MC maturation and granularity (Figure S1H).

### 2.2 The ROQUIN Paralogs Are Dispensable for MC Degranulation and Cytokine Secretion

**FIGURE 2:**
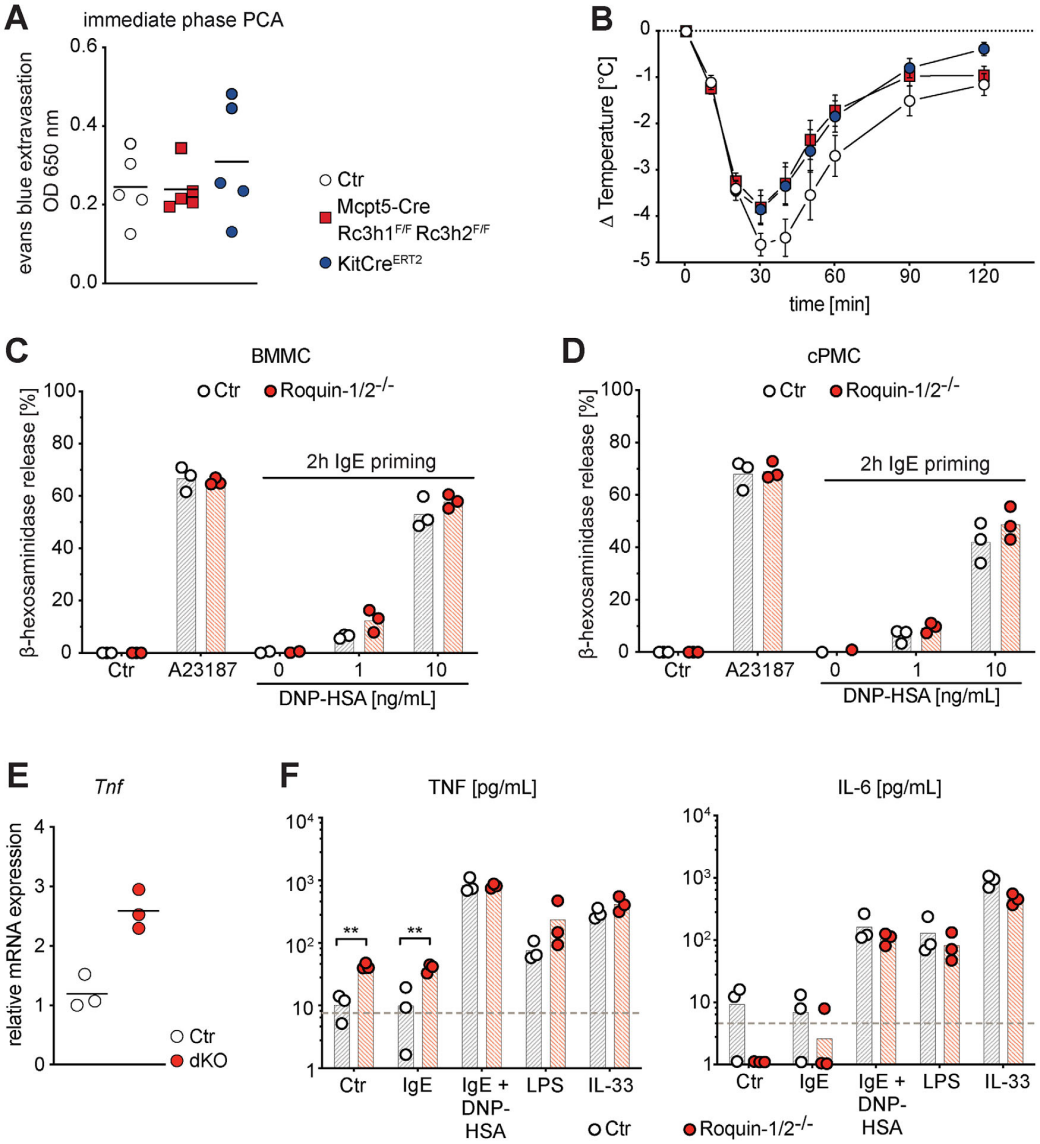
Loss of both ROQUIN paralogs does not affect MC degranulation or cytokine secretion upon activation. (A) Immediate phase passive cutaneous anaphylaxis (PCA) monitored by Evans blue extravasation. The scatter plot shows differences in dye extravasation between IgE‐ and PBS‐injected ears in five individual mice per genotype. Bars indicate medians. (B) Changes in rectal temperature over time during passive systemic anaphylaxis (PSA) reactions. Data are presented as means ± SEM (instead of SD for visual clarity) from at least nine mice per genotype. (C, D) bone marrow‐derived MCs (BMMCs) (C) or cultured peritoneal MCs (cPMCs) (D) were loaded for 2 h with 1 µg/mL anti‐DNP IgE and subsequently stimulated for 30 min with the indicated concentrations of DNP–HSA or 500 ng/mL Ca^2+^ Ionophore A23187. Degranulation was determined by measuring β‐hexosaminidase activity. % β‐hexosaminidase release corresponds to the % of activity in the supernatants of total activity (in supernatants and cell lysates). Bars represent means from three (C, D) independent MC preparations per genotype. (E) *Tnf* mRNA levels in BMMCs were determined by quantitative real‐time PCR relative to *Pbdg*. (F) TNF and IL‐6 secretion by BMMCs were determined by ELISA under the following conditions: resting, 2 h loading with 1 µg/mL anti‐DNP IgE, 2 h IgE loading, and 30 min stimulation with 10 ng/mL DNP–HSA, 6 h treatment with 10 µg/mL LPS, and 6 h treatment with 10 ng/mL IL‐33. Bars show means from three independent experiments, and the dotted line indicates the detection limit of the respective ELISA.

To evaluate the functionality of ROQUIN‐1/2 dKO MCs upon allergenic activation in vivo, we performed immediate phase passive cutaneous anaphylaxis (PCA, Figure 2A) and passive systemic anaphylaxis (PSA, Figure 2B) assays. To control for the reduced numbers and maturity of serosal MCs in Mcpt5‐Cre Rc3h1^F/F^ Rc3h2^F/F^ mice, we included heterozygous Kit^CreERT2^ knock‐in mice, which have reduced surface levels of c‐KIT, significantly reduced MC numbers in the peritoneal cavity, but normal MC numbers and location in the skin [21]. In IgE‐mediated PCA, MC‐specific ablation of ROQUIN‐1/2 did not alter vascular leakage due to antigen‐induced MC degranulation (Figure 2A). Our PSA assays showed only a trend toward a reduced drop in core body temperature in MC‐specific ROQUIN‐1/2 dKO mice comparable to Kit^CreERT2^ mice (Figure 2B), indicating that this effect arises from reduced serosal MC numbers rather than ROQUIN deficiency.

To directly evaluate MC degranulation in vitro, we employed the Kit^CreERT2^ allele to inducibly ablate ROQUIN‐1 and 2 in cultures of peritoneal (cPMC) and bone marrow‐derived (BMMC) MCs (Figure S2A,B). ROQUIN‐1/2‐deficient cPMCs and BMMCs expanded normally and did not show major alterations in the surface expression of c‐KIT, FCΕR1Α, CD16/32, and ST2 (Figure S2C). Degranulation assays revealed that ROQUIN‐1/2 dKO cPMCs and BMMCs degranulate normally in response to the Ca^2+^ Ionophore A23187 and antigen‐dependent activation following IgE loading (Figure 2C,D). In addition to the release of preformed mediators, MCs produce and secrete cytokines such as TNF and IL‐6, two prominent ROQUIN‐regulated cytokines [13, 16]. Consistently, resting ROQUIN‐1/2 dKO BMMCs had higher levels of *Tnf* mRNA and secreted more TNF protein (Figure 2E). However, upon antigen‐dependent activation, there was no difference in TNF and IL‐6 release in ROQUIN‐1/2 dKO BMMCs compared with controls (Figure 2F). Further, LPS or IL‐33‐induced TNF and IL‐6 secretion was not altered in ROQUIN‐1/2 dKO BMMCs (Figure 2F).

Therefore, in contrast to other cell types, ROQUIN‐1/2 deficiency does not enhance MC activation and functions.

### 2.3 Defining Roquin‐Mediated Posttranscriptional Gene Regulation in MCs

**FIGURE 3:**
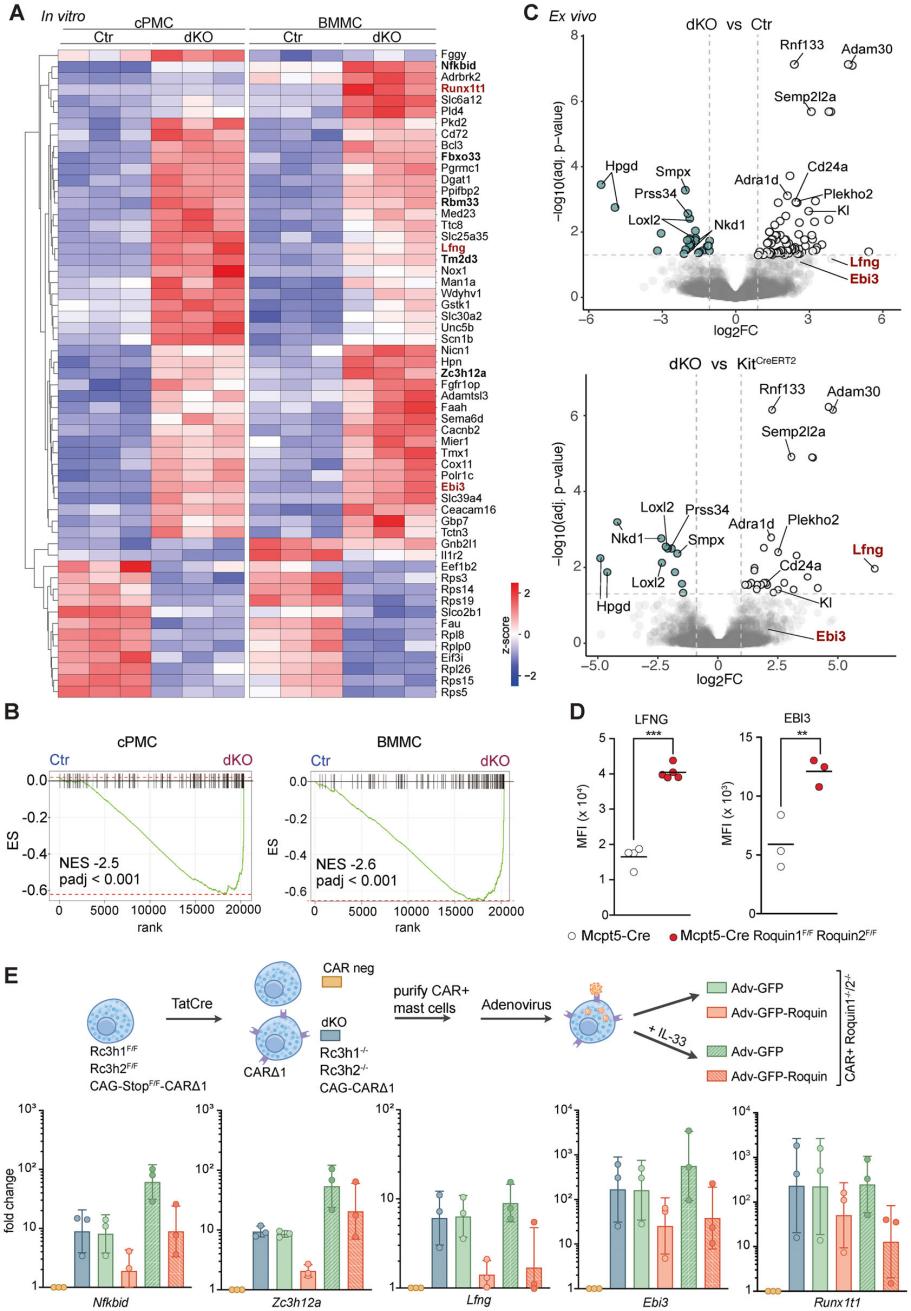
ROQUIN‐1/2 mediated posttranscriptional gene regulation in MCs. (A) The heatmap shows all common significantly altered mRNAs in vitro in three ROQUIN‐1/2 dKO cPMCs and BMMCs, compared with three respective controls (Ctr). (B) Gene set enrichment analysis (GSEA) plots of known ROQUIN target genes comparing ROQUIN‐1/2 dKO cPMCs and BMMCs with controls (Ctr). (C) Volcano plots depicting differential gene expression in ex vivo FACS‐purified peritoneal mast cells (PMCs) from five MC‐specific ROQUIN‐1/2 knockout mice compared with two control groups: four wild‐type and three Mcpt5‐Cre (Ctr), as well as four Kit^CreERT2^ mice. (D) Flow cytometry validation of increased surface expression of LFNG and EBI3 proteins in ROQUIN‐1/2 dKO primary PMCs. (E) Experimental workflow for generating CAR‐expressing ROQUIN‐1/2 dKO (CAR^+^ Roquin1^−/−^/2^−/−^) BMMCs and re‐expression of ROQUIN‐1. Real‐time PCR analysis of ROQUIN target genes under resting and IL‐33‐stimulated conditions confirms ROQUIN‐mediated repression. The graphs represent the mean ± SD from three independent experiments. Lines connect values from each experiment. Statistical significance: **p < 0.01, ***p < 0.001 (Student's *t*‐test using Welch correction).

To globally characterize ROQUIN‐mediated posttranscriptional gene regulation in MCs, we inducibly ablated ROQUIN‐1/2 in vitro in established cPMC and BMMC cultures (Figure S2A) and performed gene expression analysis. Both ROQUIN‐1/2 dKO cPMCs and BMMC cultures exhibited a consistent upregulation of known ROQUIN‐repressed transcripts, including *Nfkbid*, *Zc3h12a*, and *Tm2d3* (Figure 3A,B). We then performed gene expression analyses on ex vivo FACS‐purified primary peritoneal MCs comparing ROQUIN‐1/2 dKO to Mcpt5‐Cre and wild‐type (Ctr) as well as to Kit^CreERT2^ MCs in an attempt to control for systemic effects of ROQUIN‐1/2 deficiency, including reduced MC numbers, maturity, and c‐KIT expression [21] (Figure 3C; Figure S3D). In vitro, the cytokine subunit *Ebi3* and the RUNX1 partner transcriptional co‐repressor 1 (*Runx1t1*) were amongst the highest upregulated mRNAs in the absence of ROQUIN‐1/2 (Figure S3A), whereas ex vivo, the most significantly upregulated transcripts were *Adam30*, *Rnf133*, and *Semp2l2a* (Figure 3C). The Notch regulator lunatic fringe (*Lfng*) mRNA, which was reported to be bound by ROQUIN‐1 [13], showed increased levels in both in vitro cultured MCs and ex vivo peritoneal MCs. We validated substantially elevated LFNG protein levels in primary ROQUIN‐1/2 dKO peritoneal MCs by flow cytometry (Figure 3C,D). EBI3 protein levels were also upregulated in primary ROQUIN‐1/2 dKO PMCs (Figure 3D), although ex vivo, the upregulation of its mRNA did not reach significance. On the other hand, the unchanged expression of *Adam30*, *Rnf133*, and *Semp2l2a* in the absence of ROQUIN‐1/2 in mast cell cultures in vitro suggests that their elevated expression ex vivo stems most likely from the immature maturation state of peritoneal MCs in Mcpt5‐Cre Rc3h1^F/F^ Rc3h2^F/F^  mice.

To further validate our in vitro gene expression dataset and to assess ROQUIN‐mediated repression more directly, we acutely ablated both ROQUIN paralogs in BMMCs through Cre protein transduction with concurrent CAR expression (Figure 3E). In the resulting CAR^+^ ROQUIN‐1/2 dKO BMMCs, we then re‐expressed ROQUIN‐1 together with eGFP through adenoviral transduction, using eGFP re‐expression in these cells as controls. The influence of MC activation was assessed by IL‐33 treatment. Transcript abundance changes of *Ebi3* and *Runx1t1* mirrored closely those of the known ROQUIN targets *Nfkbid* and *Zc3h12a*, as well as the ROQUIN‐1‐bound *Lfng* (Figure 3E), confirming their ROQUIN‐mediated repression in MCs. To test direct regulation of *Ebi3*, *Lfng*, and *Runx1t1* mRNAs by ROQUIN‐1/2, we generated and validated stable expression of dual fluorescent 3'‐UTR reporter constructs in murine embryonic fibroblasts, allowing inducible ablation of ROQUIN‐1/2 (Figure S4A,B). Induced loss of ROQUIN‐1/2 did not affect *Ebi3* and *Runx1t1* 3'‐UTR‐controlled reporter expression, suggesting that the ROQUIN‐mediated regulation of their expression is indirect. Fitting to the presence of ROQUIN binding sites in its 3′‐UTR, the fluorescent output from a GFP mRNA fused to the *Lfng* 3'‐UTR increased by 1.8‐fold upon induced ablation of ROQUIN‐1/2, indicating direct regulation (Figure S4C).

Collectively, our experiments globally define transcripts affected by ROQUIN‐1/2 ablation in MCs, including indirect repression of *Ebi3* and *Runx1t1* as well as direct repression of *Lfng* via its 3′‐UTR.

### 2.4 Data Limitations and Perspectives

In our in vitro and ex vivo transcriptomic profiling, we did not identify candidate mechanisms to explain the near‐complete loss of mature MCs in the serous cavities of MC‐specific ROQUIN‐1/2–deficient mice. We identified a reduced sensitivity of ROQUIN‐1/2–deficient MCs to survival signals in vitro (Figure S5). However, in the absence of an in vitro model that recapitulates developmental and survival signals specific to serosal MCs, we are unable to functionally link this finding or specific ROQUIN targets to the in vivo phenotype. Consequently, the mechanisms by which ROQUIN paralogs mediate the differentiation and/or maintenance of serosal MCs remain to be uncovered in future studies. Furthermore, although passive systemic anaphylaxis experiments suggest that MC functionality is largely intact, we cannot rule out further MC deficiencies in individual organs in MC‐specific ROQUIN1/2‐deficient mice.

### 2.5 Concluding Remarks

We show that MC‐specific ROQUIN paralog ablation leads to loss of mature MCs in the peritoneal and pleural cavities, while skin MC populations remain unaffected. This highlights specific requirements for the development and/or maintenance of MCs in serous cavities, differing from MCs in other locations.

## 3 Experimental procedures

### 3.1 Genetically Modified Mice

Mcpt5Cre [22], Kit^CreERT2^ [23], R26/CAG‐Stop^F^‐CAR1 [20], Rc3h1^F/F^ [24], Rc3h2^F/F^ [25], Mcpt5Cre Rc3h1^F/F^, Mcpt5Cre Rc3h2^F/F^ and Mcpt5Cre Rc3h1^F/F^ Rc3h2^F/F^ and Kit^CreERT2^ Rc3h1^F/F^ Rc3h2^F/F^ mice were kept on a C57BL/6 genetic background. All the mice used in this study were housed under specific pathogen‐free (SPF) conditions, according to the legislation of the European Union and the Region of Upper Bavaria. Mice were bred and housed in the animal facilities of the Max Planck Institute of Biochemistry and at the Centre for Preclinical Research of the MRI (Zentrum für Präklinisches Forschung [ZPF], Munich). Standard food was available ad libitum for all animals. For all genotypes, sex‐matched female or male mice (aged 7 to 15 weeks) were used in the experiments.

### 3.2 Flow Cytometry

At least 10^5^ cells per sample were stained and kept on ice. Antibodies and cells were diluted in PBS supplemented with 0.5% BSA, 2 mM EDTA, and 0.1% sodium azide (FACS buffer). Nonspecific binding of antibodies to isolated single cells was minimized by incubation with Fc‐block solution (anti‐CD16/32, clone 93; eBioscience) at 4°C for 15 min. Next, cells were stained for 30 min at 4°C with primary antibodies and washed once with FACS buffer. Dead cells were excluded by staining with 7‐aminoactinomycin D (7‐AAD; eBioscience) or ethidium monoazide (EMA; Invitrogen). If primary antibodies were unlabeled, cells were stained with fluorescently labeled secondary antibodies for 30 min. For intracellular stainings, cells were fixed and permeabilized using the Foxp3/Transcription Factor Staining Buffer Set (eBioscience, 60 min, 4°C). The following monoclonal antibodies were used for surface or intracellular staining: anti‐c‐Kit (2B8), anti‐FCERIA (MAR‐1), anti‐CD16/32 (clone 93) (all from eBioscience), anti‐CAR (E1‐1; Santa Cruz Biotechnology), anti‐LFNG (EPR10391(B); Abcam), anti‐EBI3 (#355022; R&D Systems), and anti‐ST2 (T1/ST2) (all from BioLegend). Flow cytometry data were acquired on a Cytoflex LX (BC), FACSCalibur, FACSCanto II, or cells were sorted using a FACSAria II flow cytometer (all from BD Biosciences). Data were analyzed using FlowJo software (Tree Star).

### 3.3 Immunofluorescence and Immunohistology

For whole‐mount ear skin immunofluorescence microscopy, ears were separated into dorsal and ventral sheets, and cartilage‐free ear sheets were fixed by floating on 1% formaldehyde overnight at 4°C. Ear sheets were blocked with 1% BSA and stained with a rabbit anti‐laminin antibody (gift from Michael Sixt), followed by Cy3‐conjugated anti‐rabbit (Jackson ImmunoResearch), and FITC‐conjugated avidin (Zymed). Images were acquired with a confocal laser‐scanning microscope (Zeiss LSM 780) or a fluorescent microscope (Zeiss AxioImager Z1), and the images were analyzed using ImageJ. For cytospins, cells from peritoneal lavages were centrifuged onto glass slides, air dried, and stained with toluidine blue (Sigma‐Aldrich). For back skin immunohistology, dorsal skin was fixed with 4% paraformaldehyde, paraffin‐embedded, sectioned (10 µm), and stained with toluidine blue or safranin and alcian blue (all Sigma‐Aldrich).

### 3.4 Passive Cutaneous and Systemic Anaphylaxis

For immediate phase PCA reactions, mice were passively sensitized by intradermal injection of 100 ng anti‐DNP IgE into one ear and PBS into the contralateral ear. After 24 h, mice were challenged by intravenous (i.v.) injection of 200 µg DNP‐HSA in 0.5% Evans blue (both Sigma‐Aldrich). Extravasation was quantified by dimethylformamid extraction and photometric quantification. For PSA reactions, mice were sensitized by intraperitoneal (i.p.) injection of 10 µg anti‐DNP IgE and 24 h later challenged by i.p. injection of 100 µg DNP‐HSA. Systemic anaphylactic response was monitored by measuring changes in body temperature using a rectal thermometer (Bioseb). Anti‐DNP specific IgE (clone SPE7) was produced by SPE‐7 hybridoma NS1 cells.

### 3.5 MC In Vitro Cultures

Bulk bone marrow and peritoneal cells were cultured in suspension using MC medium to generate bone marrow‐derived mast cells (BMMCs) and cultured peritoneal mast cells (cPMCs). The MC medium was composed of Dulbecco's Modified Eagle Medium (DMEM; Gibco) supplemented with 10% heat‐inactivated fetal calf serum (FCS; Sigma‐Aldrich), 2% supernatant from X63/0 myeloma cells expressing interleukin‐3 (IL‐3) (kindly provided by Ton Rolink), and 0.5% supernatant from CHO cells expressing stem cell factor (SCF) (gift from Patrice Dubreuil). The medium was further supplemented with GlutaMAX (Gibco), nonessential amino acids (Gibco), 50 µM 2‐mercaptoethanol (Merck), and 25 mM HEPES buffer (Gibco). Cells were maintained under standard culture conditions (37°C, 5% CO_2_) for a period of four weeks to allow for MC differentiation.

Four weeks after the initiation of MC cultures, the knockout of ROQUIN‐1, ROQUIN‐2, or both ROQUIN paralogs, as well as the induction of CAR expression in cKit^CreERT2^‐containing BMMCs and cPMCs with or without conditional *Rc3h‐½* and *R26/CAG‐Stop^F^‐CAR1* alleles, was achieved by supplementing the MC medium with 1 µM 4‐hydroxytamoxifen (4‐OHT; Sigma‐Aldrich) for 7 consecutive days.

For Cre‐mediated recombination using His‐Tat‐NLS‐Cre (HTNC or Tat‐Cre), single‐cell suspensions of MCs were washed with PBS and incubated at 37°C in serum‐free medium (HyClone, Thermo Scientific) containing 2 µM HTNC and 50 µg/mL polymyxin B (Calbiochem), 200 µM chloroquine, and 2 µM leupeptin (all from Sigma‐Aldrich) for 5 h.

### 3.6 Mast Cell Degranulation Assays and ELISAs

To induce degranulation, BMMCs and cPMCs were sensitized for 2 h with 1 µg/mL anti‐DNP IgE (SPE‐7 hybridoma supernatant). After washing in Tyrode's buffer (10 mM HEPES, pH 7.3, 135 mM NaCl, 5 mM KCl, 1.8 mM CaCl_2_, 1 mM MgCl_2_, 5.6 mM glucose, 0.5 mg/mL BSA), cells were stimulated with the different concentrations of DNP–HSA indicated in the graphs or 500 ng/mL A23187 for 30 min. β‐hexosaminidase activity in supernatants and cell pellets, which were solubilized with 0.5% Triton X‐100 in Tyrode's buffer, was measured using p‐nitrophenyl‐N‐acetyl‐β‐d‐glucosaminide (Sigma‐Aldrich).

The levels of TNF (PeproTech) and IL‐6 (BD) were determined by ELISA according to the manufacturer's instructions.

### 3.7 Gene Expression Analysis

Quantitative real‐time PCR: Total RNA from FACS‐purified primary MCs and cultured MCs was isolated and reverse‐transcribed (Promega) for quantitative real‐time PCR using Universal Probe Library (Roche Diagnostics) probes and primers according to the instructions of the manufacturer.

Affymetrix gene array of in vitro cultured MCs, Agilent gene array of ex vivo FACS‐purified 7‐AAD‐negative cKIT^+^ ST2^+^ primary MCs from peritoneal lavages of 3 Mcpt5‐Cre, 5 Mcpt5‐Cre Rc3h1^F/F^ Rc3h2^F/F^, 4 wild‐type, and 4 Kit^CreERT2/+^ mice (performed by Miltenyi), and gene expression analyses are detailed in the supplementary materials and methods.

### 3.8 Adenoviral Infections

Adenovirus production and transduction were conducted as previously described [20]. Briefly, single MC suspensions were incubated with concentrated adenoviral lysates at a multiplicity of infection (MOI) of 10 for 3 h at 37°C. Following infection, the cells were washed three times with PBS and then cultured in the MC medium until further analysis.

## Author Contributions

Designed and performed experiments: K.H., A.M., C.L., A.V.L., S.B., T.Z. Analyzed and interpreted data: K.H., A.M., A.Y., L.O., T.Z., D.K., M.S.S. Wrote the paper: M.S.S., A.M., D.K.

## Ethics Statement

All animal experiments were performed in compliance with both European Union and German law and approved by local authorities (Regierungspräsidium Oberbayern, 55.2‐1‐54‐2532‐150‐12, 55.2‐1‐54‐2532‐234‐2015).

## Conflicts of Interest

The authors declare no conflicts of interest.

## Supporting information




**Supporting File 1**: eji70110‐sup‐0001‐FiguresS1‐S5.pdf.

**Supporting File 2**: eji70110‐sup‐0002‐GatingStra.pdf.

**Supporting File 3**: eji70110‐sup‐0003‐SuppMat.xlsx.

**Supporting File 4**: eji70110‐sup‐0004‐SuppMat.xlsx.

**Supporting File 5**: eji70110‐sup‐0005‐SuppMM.pdf.

## Data Availability

The Affymetrix gene array datasets (GSE295430) and the Agilent gene array datasets (GSE294451) were deposited at GEO.
