# Supplementary material for: Serous Cavity Mast Cells Depend on the ROQUIN Paralogs
Source: Eur J Immunol. 2025 Dec 19;55(12):e70110. doi: 10.1002/eji.70110 (PMC12716222; doi:10.1002/eji.70110)
Supplement: Supplementary file 1 — Supporting File 1: eji70110‐sup‐0001‐FiguresS1‐S5.pdf. [file EJI-55-e70110-s002.pdf]

Supplementary figures

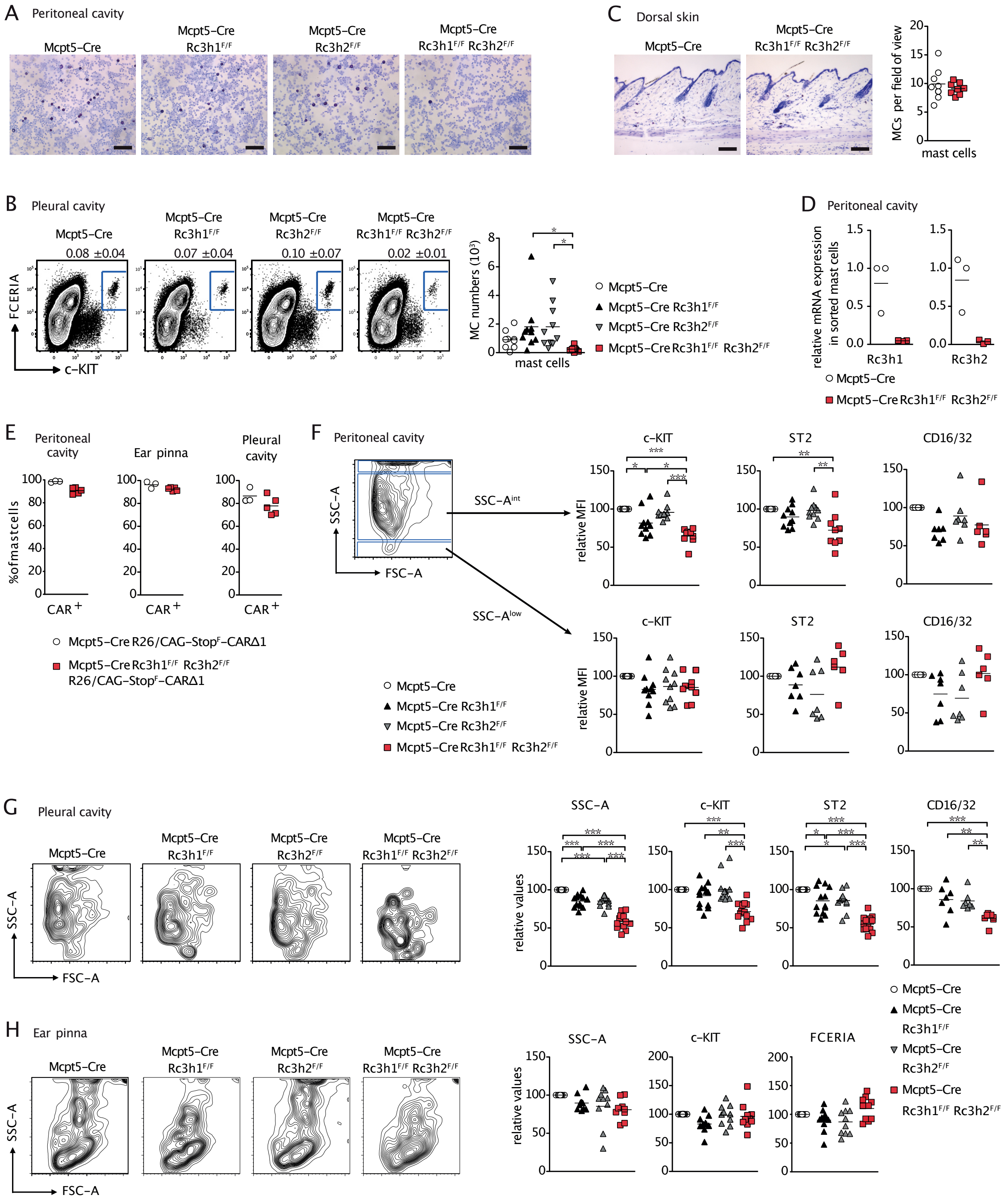

Figure S1. Phenotypic characterization of *ex vivo* isolated MCs.

(A) Representative images of Toluidine blue stained cytospins from peritoneal cavity lavages of mice (three per genotype). Scale bar: 100 µm.

(B) Flow cytometry plots showing proportions of pleural cavity MCs. The numbers above the plots represent mean MC frequencies ± SD from at least 10 mice per genotype. The Scatter plot to the right displays absolute MC numbers.

(C) Representative images of dorsal skin sections stained with Toluidine blue. Scale bar: 100 µm. Scatter plot shows MC frequencies; each data point represents the mean MC count across ten fields of view per mouse (n = 8 per genotype).

(D) Scatter plots showing relative mRNA levels of *Rc3h1* and *Rc3h2* in FACS-purified primary peritoneal MCs, measured by quantitative real-time PCR (3 mice per genotype).

(E) Percentage of CAR^+ MCs of total MCs in the peritoneal cavity, ear pinna, and pleural cavity of mice of the indicated genotypes. Expression of CAR is induced by Cre and indicates efficient Cre-mediated recombination in the cells.

(F) Relative expression levels (MFI) of c-KIT, ST2, and CD16/32 on SSC-A^{int} and SSC-A^{low} peritoneal MCs measured by flow cytometry. The values for MCs from MC-specific ROQUIN-1 KO, ROQUIN-2 KO and ROQUIN-1/2 dKO mice were normalized to corresponding MCs from Mcpt5-Cre control mice (at least 8 per genotype).

(G) Flow cytometry plots showing forward scatter (FSC-A) and side scatter (SSC-A) properties of pleural cavity MCs of the indicated genotypes. Corresponding scatter plots display relative MFI for SSC-A, c-KIT, ST2, and CD16/32 in each genotype, normalized to controls as in (F) (at least 8 per genotype).

(H) Flow cytometry plots of ear pinna MCs showing FSC-A and SSC-A properties. Scatter plot indicates relative MFI for SSC-A, c-KIT, and FCERIA, normalized to controls (at least 8 per genotype).

*$p<0.05$, **$p<0.01$, ***$p<0.001$ (Student's t-test with Welch correction or one-way ANOVA).

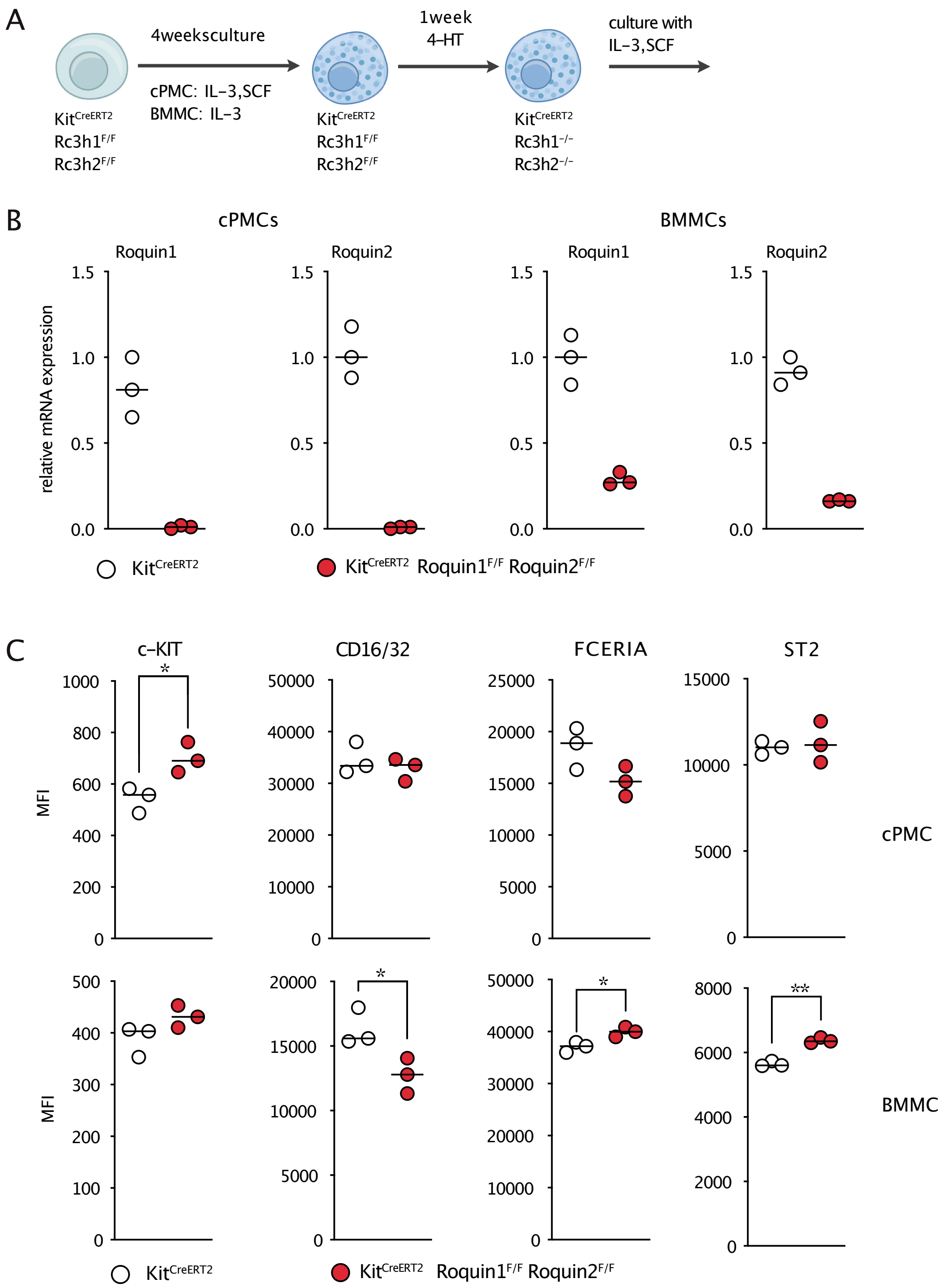

Figure S2. Validation of ROQUIN-1/2 ablation and MC phenotype *in vitro*.

(A) Schematic of the experimental workflow for generating ROQUIN-1/2 double-knockout (dKO; Rc3h-$1^{-/-}/2^{-/-}$) MC *in vitro*.

(B) Scatter plots showing mRNA levels of *Rc3h1* and *Rc3h2* relative to *Pbdg* determined by quantitative real-time PCR, confirming efficient Cre-mediated gene inactivation in both cPMCs and BMMCs.

(C) Scatter plots of mean fluorescence intensity (MFI) for MC-related surface markers in cPMCs and BMMCs. *$p < 0.05$, **$p < 0.01$ (Student's t-test with Welch's correction).

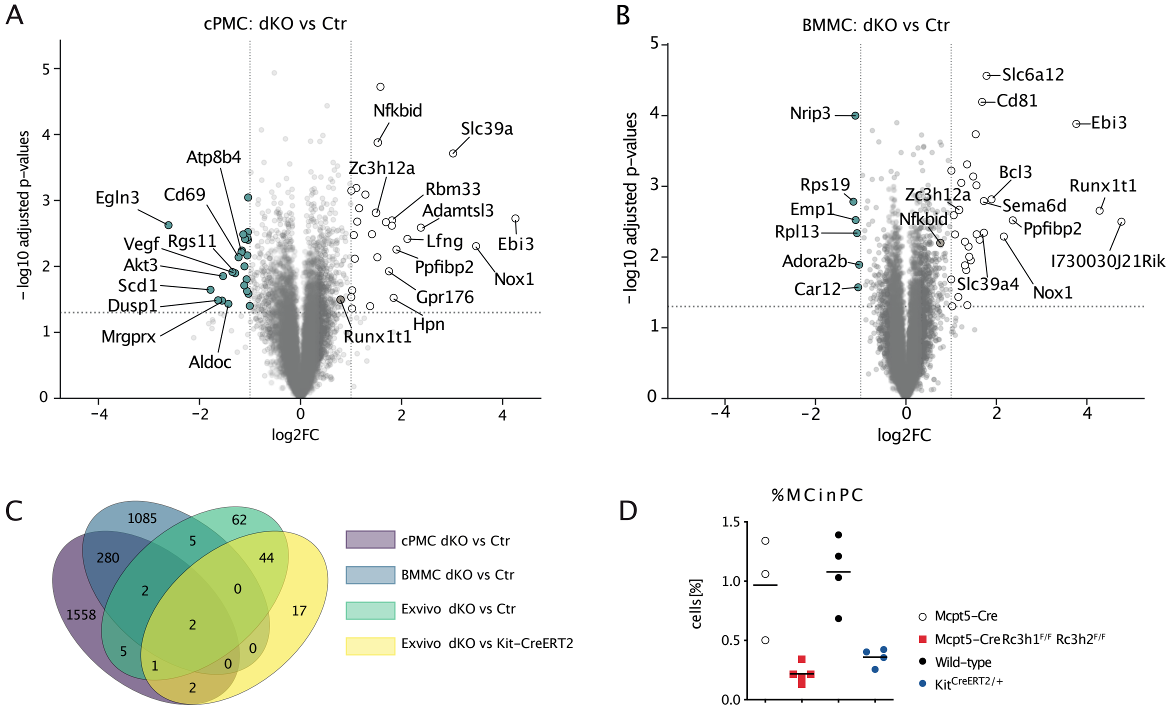

Figure S3. Additional analyses of ROQUIN-1/2 dKO MCs and validation of gene expression changes.

(A, B) Volcano plots showing differential gene expression profiles in ROQUIN-1/2-deficient (Rc3h1$^{F/F}$ Rc3h2$^{F/F}$ MCs treated for one week with 4-hydroxytamoxifen as shown in S2A): cPMCs (A) and BMMCs (B) compared to c-Kit^{CreERT2} controls.

(C) Four-way Venn diagrams illustrate the overlap of differentially regulated transcripts across multiple comparisons, including ROQUIN-1/2 dKO versus control cPMC, ROQUIN-1/2 dKO versus control BMMC, *ex vivo* isolated ROQUIN-1/2 dKO versus control MCs, and *ex vivo* isolated ROQUIN-1/2 dKO versus Kit-CreERT2 control MCs. The list of commonly dysregulated transcripts identified in all four datasets is provided in Table S2.

(D) Scatter plot showing the percentage of MCs in peritoneal cavity lavage samples used for *ex vivo* gene expression analysis.

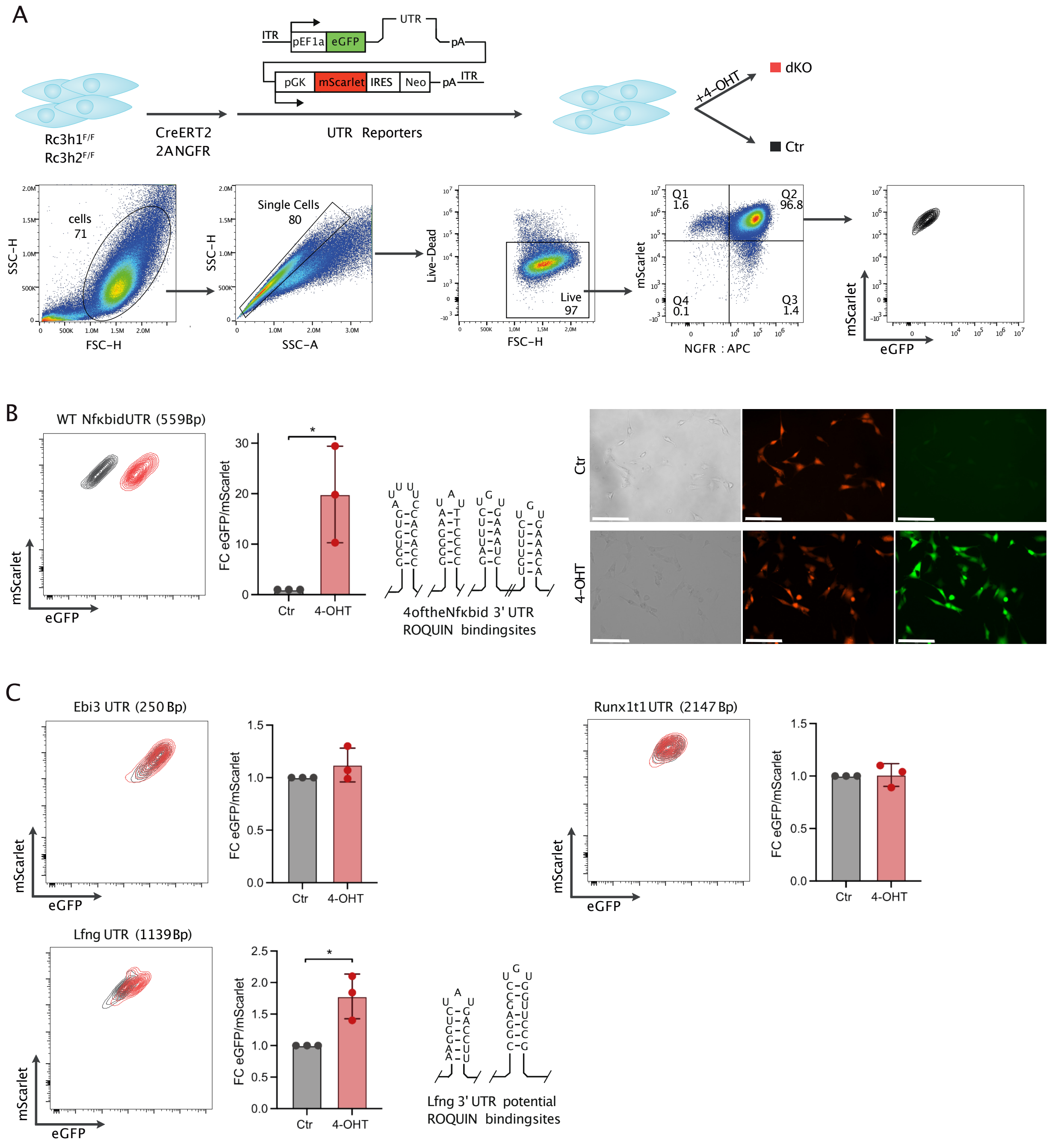

Figure S4. Dual fluorescent 3' UTR reporter assays for the candidate Roquin-regulated mRNAs *Ebi3*, *Lfng,* and *Runx1t1*

(A) Schematic representation of the experimental workflow used to generate Rc3h1$^{F/F}$/2$^{F/F}$ mouse embryonic fibroblasts (MEFs) stably expressing CreERT2 and 3' untranslated region (3' UTR) reporter constructs. Rc3h1$^{F/F}$/2$^{F/F}$ MEFs were first transduced with a retrovirus encoding CreERT2 and NGFR, enabling both tamoxifen-inducible ablation of

ROQUIN-1 and ROQUIN-2 (dKO) upon 4-hydroxytamoxifen (4-OHT) treatment and identification of transduced cells via NGFR expression. Dual fluorescent UTR reporter constructs (Riess et al.) were cloned and subsequently introduced using the PiggyBac transposon system. Each UTR reporter construct contained mScarlet expressed under the control of a PGK promoter, to confirm genomic integration, and eGFP fused to the 3' UTR of putative ROQUIN target genes under control of an EF1α promoter. PiggyBAC transposase inverted terminal repeat sequences (ITR) facilitate genomic integration of the ITR-flanked sequences when PiggyBAC is co-electroporated and an IRES-neomycin resistance gene sequence allows selection of reporter expressing cells. Changes in the ratio of eGFP to mScarlet fluorescences indicate UTR regulation. Changes induced by ablation of ROQUIN-1 and 2 indicate Roquin-mediated regulation of the UTR. Representative flow cytometry gating strategies are shown: after gating on live single cells, $NGFR^+$(CreERT2-expressing) and $mScarlet^+$(UTR reporter-expressing) cells were selected for subsequent analysis.

(B) The *Nfkbid* 3' UTR, which contains multiple validated ROQUIN binding sites (Essig et al.), was used as a positive control. Upon 4-OHT-induced ablation of ROQUIN-1/2, a pronounced increase in GFP fluorescence was observed in MEFs carrying the *Nfkbid* UTR, indicating loss of ROQUIN-1/2-mediated suppression. Fluorescence microscopy analysis supports the flow cytometry results, showing increased GFP signal intensity in 4-OHT–treated MEFs (dKO) compared to untreated controls. Cells expressing the *Nfkbid* 3′UTR reporter displayed robust cytoplasmic GFP accumulation upon ROQUIN-1/2 ablation. (scale bar = 150 µm).

(C) Representative flow cytometry contour plots and corresponding quantification for reporter constructs containing the 3′UTRs of *Ebi3*, *Lfng*, and *Runx1t1* are shown. Using unafold, we identified putative ROQUIN binding sites only in the *Lfng* 3' UTR. $*p < 0.05$, $**p < 0.01$ (Student's t-test with Welch's correction).

Rieß, David Karl (2017): The role of Roquin proteins in B cell physiology and pathology. Dissertation, LMU München: Fakultät für Chemie und Pharmazie

Essig, K., Kronbeck, N., Guimaraes, J.C. et al. Roquin targets mRNAs in a 3′-UTR-specific manner by different modes of regulation. Nat Commun 9, 3810 (2018).

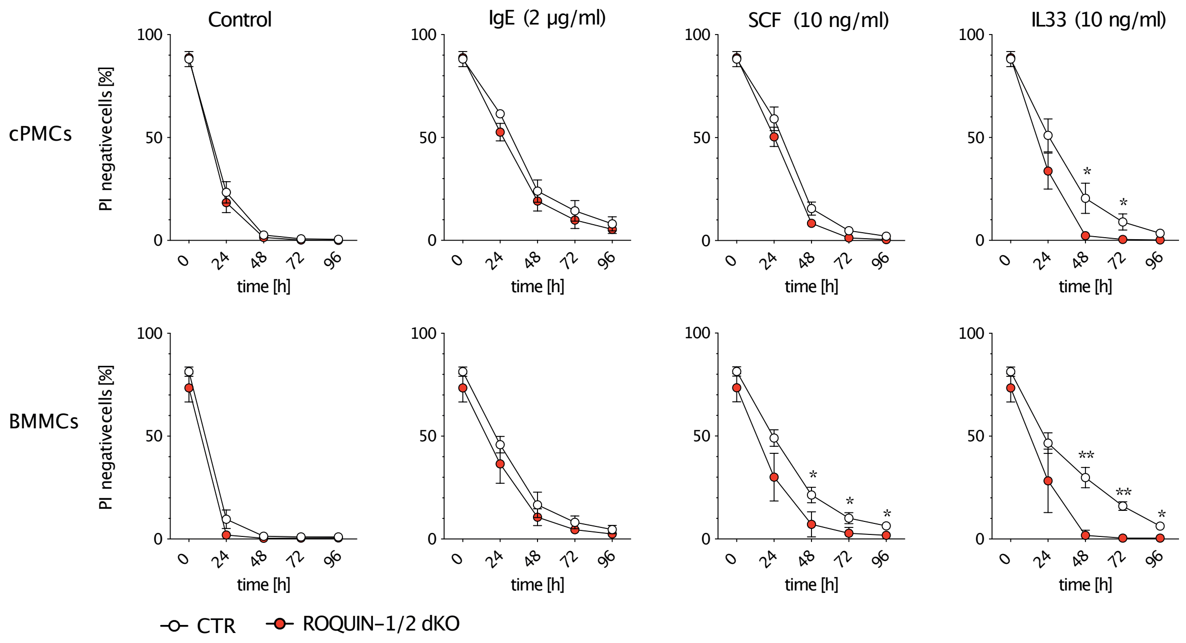

Figure S5. ROQUIN-1/2-deficient MCs exhibit decreased sensitivity to survival signals.

To assess the sensitivity of ROQUIN-1/2-deficient MCs to growth factor withdrawal-induced cell death in comparison to controls (c-KitCreERT2), cPMCs and BMMCs were cultured under serum-starving conditions (0.5 % FCS) without IL-3 or SCF for 96 h. The proportion of live (propidium iodide-negative) cells was determined by flow cytometry at the indicated time points. A dramatic reduction in cell viability was already observed after 24 h in both cPMCs and BMMCs. Supplementation of the culture medium with survival promoting factors, including IgE, SCF, and IL-33, reduced cell death, especially in WT cells. The difference between control and ROQUIN-1/2 dKO cPMCs and BMMCs was most pronounced in IL-33–treated cultures. *$p < 0.05$, **$p < 0.01$ (Student's t-test with Welch's correction). Data are shown as mean values of 3 independent mast cell preparations and error bars indicate standard deviations. The BMMC data are representative of 3 independent experiments.
