# Supplementary material for: Serous Cavity Mast Cells Depend on the ROQUIN Paralogs
Source: Eur J Immunol. 2025 Dec 19;55(12):e70110. doi: 10.1002/eji.70110 (PMC12716222; doi:10.1002/eji.70110)

Gating Strategies

Fig. 1A, B, E, F and S1B, E, F,G: PMCs

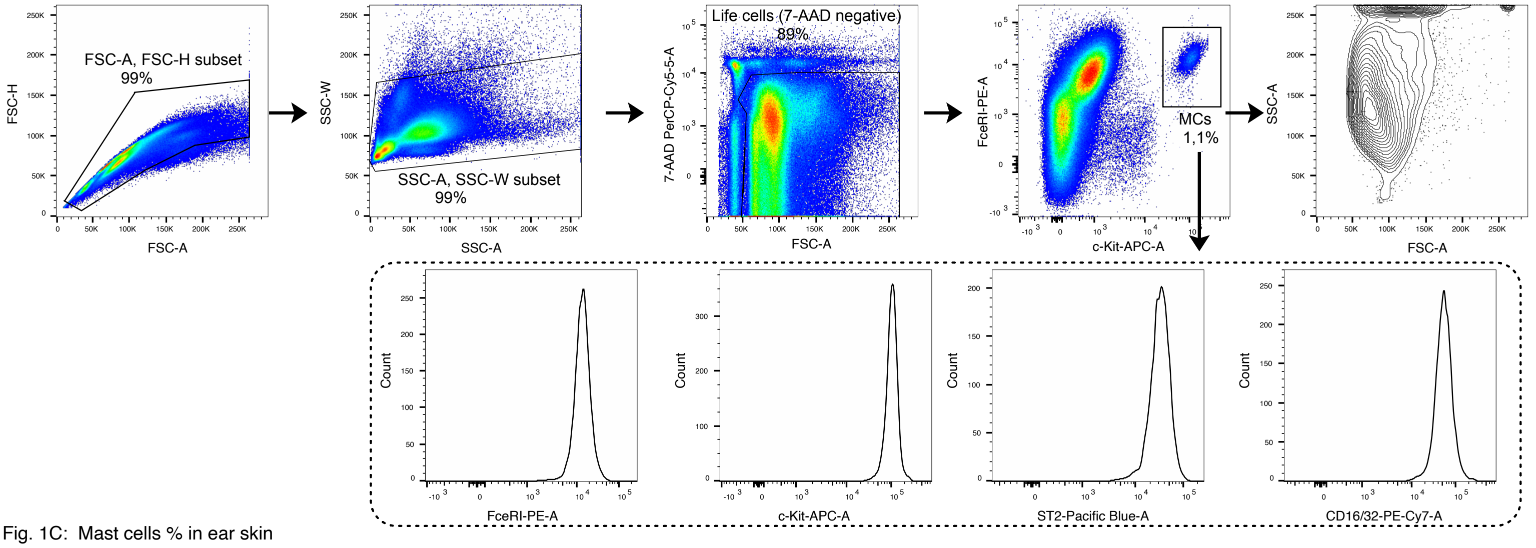

Fig. 1C: Mast cells % in ear skin

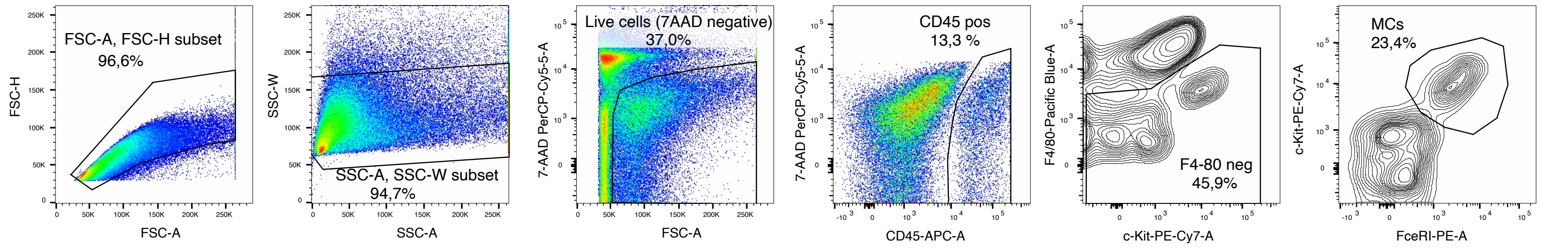

Fig. 3C, S3C: PMCs FACS purification for *ex vivo* gene expression analysis

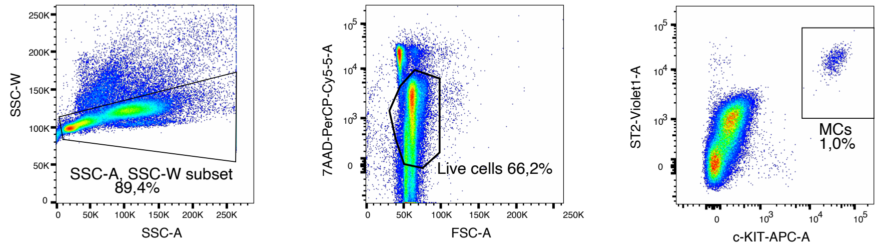

Fig. 3D: Validation in primary PMCs

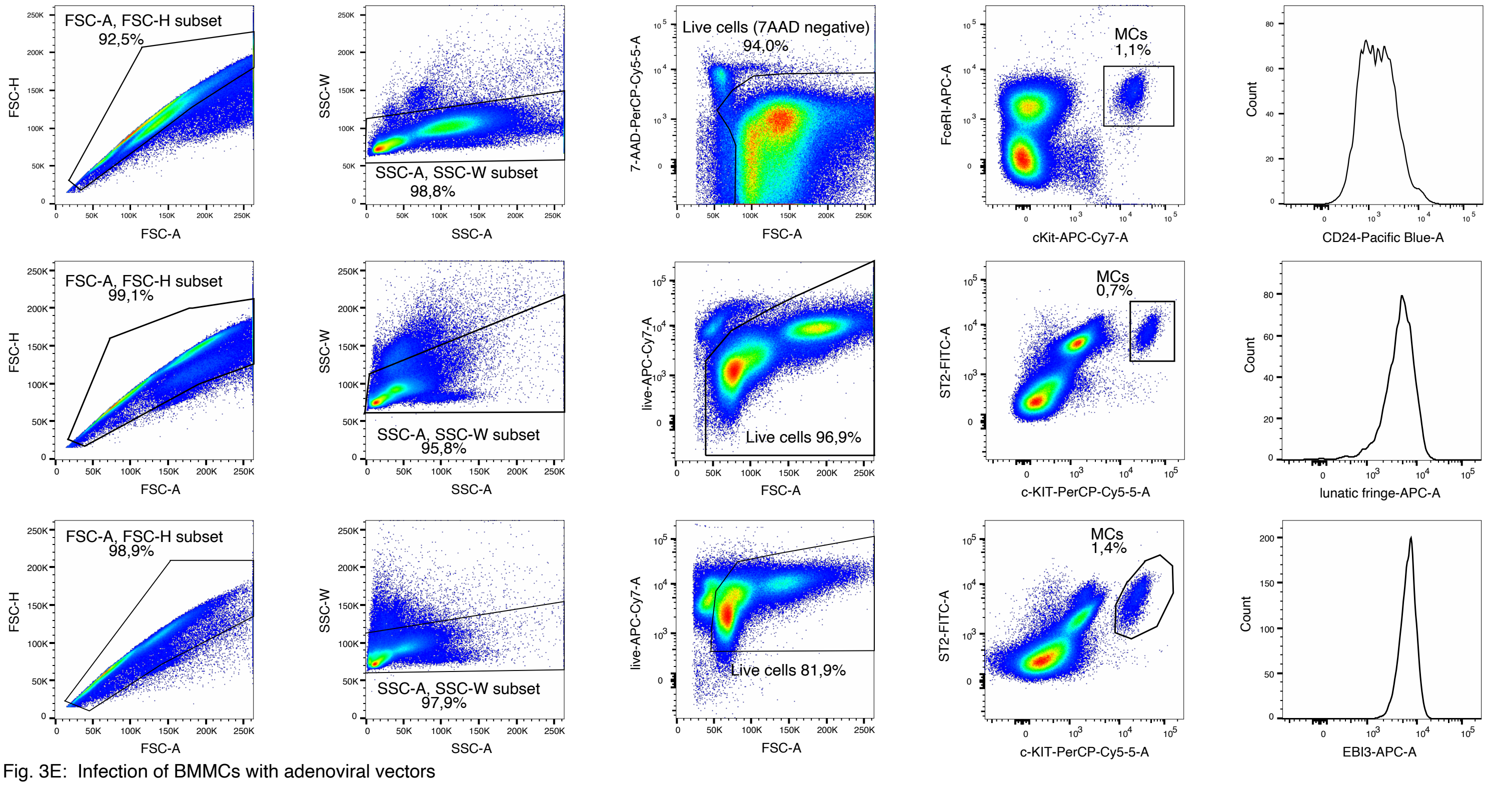

Fig. 3E: Infection of BMMCs with adenoviral vectors

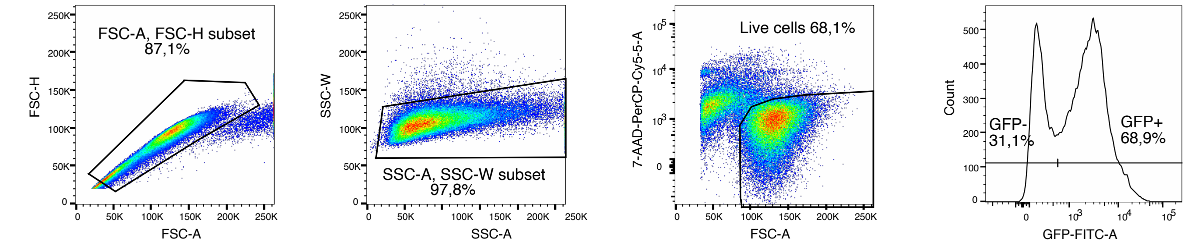

Supplement: Supplementary file 2 — Supporting File 2: eji70110‐sup‐0002‐GatingStra.pdf. [file EJI-55-e70110-s005.pdf]
