# Supplementary material for: Serous Cavity Mast Cells Depend on the ROQUIN Paralogs
Source: Eur J Immunol. 2025 Dec 19;55(12):e70110. doi: 10.1002/eji.70110 (PMC12716222; doi:10.1002/eji.70110)
Supplement: Supplementary file 5 — Supporting File 5: eji70110‐sup‐0005‐SuppMM.pdf. [file EJI-55-e70110-s001.pdf]

Supplementary Materials and Methods

Gene expression analysis

Affymetrix gene array of *in vitro* cultured MCs: Total RNA from three cPMC and three BMMC preparations was extracted using a Qiagen RNAeasy Micro kit. 50 ng of total RNA were used for biotin labeling (Affymetrix GeneChip whole transcript sense target-labeling kit) and hybridized to Affymetrix M430 V2 microarrays. Array normalization and expression value calculation was performed using DNA-Chip Analyzer (www.dchip.org). Data visualization, including heatmaps and volcano plots, was performed using Python (version 3.9.2). Gene set enrichment analysis (GSEA) was conducted using either the fgsea (1) package or the clusterProfiler package (version 4.14.3) in R (2). For GSEA, genes were ranked based on the Wald test statistic (*stat*), and a baseMean threshold of >2 was applied to filter for sufficiently expressed genes before enrichment analysis. Agilent gene array (performed by Miltenyi Biotech) of *ex vivo* FACS-purified primary MCs: Primary PMCs were isolated directly *ex vivo* by FACS-based purification of 7-AAD-negative $cKIT^{+}$ $ST2^{+}$ MCs from peritoneal lavages of 16 (3 Mcpt5-Cre, 5 Mcpt5-Cre $Rc3h1^{F/F}$ $Rc3h2^{F/F}$, 4 wild-type, 4 $Kit^{CreERT2/+}$) mice. The FACS-purified murine MCs were lysed using SuperAmpTM Lysis Buffer and shipped to Miltenyi Biotech.

The differential gene expression analysis was performed using the limma package in R (3). Raw data of Agilent and Affymetrix microarrays were preprocessed, including background correction using normexp for Agilent and RMA for Affymetrix, followed by quantile normalization and log2-transformation to stabilize variance across samples. A linear model was fitted to the expression data, and empirical Bayes moderation was applied to borrow information across genes, improving the robustness of variance estimates for datasets with limited replicates. Differential expression was assessed using moderated t-tests, with significance defined as an adjusted p-value (FDR) < 0.05 and an absolute log2 fold change > 1. Results were visualized using volcano plots (generated with ggplot2 (v. 3.5.1), where log2 fold changes were plotted against statistical significance (-log10-adjusted p-values). Custom themes and color scales enhanced the aesthetics for publication quality.

Four-way Venn Analysis

Gene lists from ROQUIN-1/2 dKO versus CTR cPMC, ROQUIN-1/2 dKO versus CTR BMMC, *ex vivo* isolated ROQUIN-1/2 dKO versus CTR MC, and *ex vivo* isolated ROQUIN-1/2 dKO versus Kit-creERT2 MC comparisons were processed and analyzed in Python using the pandas, matplotlib, and venn libraries. Region membership tables were optionally exported with openpyxl.

Dual-fluorescence 3' UTR reporter assays

Rc3h1$^{F/F}$ Rc3h2$^{F/F}$ mouse embryonic fibroblasts (MEFs) were engineered to stably express CreERT2 and 3' UTR reporter constructs. MEFs were first transduced with a retroviral vector encoding CreERT2 and nerve growth factor receptor (NGFR). NGFR-positive cells were FACS-purified, enabling tamoxifen-inducible ablation of *Rc3h1* and *Rc3h2* dKO upon treatment with 0.2 µM 4-hydroxytamoxifen (4-OHT) (Sigma-Aldrich).

Dual-fluorescent 3' UTR reporter constructs (*Nfkbid, Lfng, Ebi3, Runx1t1)* were cloned and stably integrated into the genome using the PiggyBac transposon system. Each construct contained mScarlet, driven by a PGK promoter, serving as a marker for successful genomic integration and internal fluorescent reference, and enhanced GFP (eGFP) under control of an EF1α promoter, fused to the 3' UTRs of candidate Roquin target genes. PiggyBac inverted terminal repeat (ITR) sequences enabled stable integration when co-electroporated with PiggyBac transposase, while an IRES–neomycin resistance cassette allowed selection and enrichment of stable transfectants using G418 (300 µg/ml).

Cells were treated with 4-OHT–containing medium for 24 hours, then the medium was replaced with fresh, complete medium, and the cells were maintained under normal conditions for an additional 3 days to allow for complete loss of ROQUIN-1 and ROQUIN-2 proteins prior to subsequent assays. Cells were trypsinized, harvested, and stained with Live/Dead dye (iFluor® 840 maleimide, AAT Bioquest) and APC–anti-NGFR antibody (BioLegend). Sequential gating was applied to select live single cells, followed by NGFR^{+} (CreERT2-expressing) and mScarlet^{+} (UTR reporter–expressing) populations. Mean fluorescence intensities (MFI) of eGFP and mScarlet were quantified using FlowJo, and the eGFP-to-mScarlet fluorescence ratio was calculated to assess post-transcriptional

regulation mediated by the respective 3' UTRs. Changes in this ratio following 4-OHT–induced ablation of ROQUIN-1 and ROQUIN-2 were interpreted as evidence of Roquin-dependent regulation of the tested UTRs.

Survival and Sensitivity to Growth Factor Withdrawal

To evaluate the effect of ROQUIN-1 and ROQUIN-2 deficiency on mast cell survival under growth factor–limited conditions, cPMCs and BMMCs were generated and depleted of ROQUIN-1/2 as described above, and after differentiation, subjected to serum starvation. Cells were cultured in DMEM containing 0.5% fetal calf serum (FCS) without IL-3 or SCF to induce apoptosis, and also supplemented with IgE (2 µg/ml), SCF (10 ng/ml), or IL-33 (10 ng/ml). Cell viability was assessed at multiple time points for up to 96 hours using flow cytometry.

References:

1. Sergushichev AA. An algorithm for fast preranked gene set enrichment analysis using cumulative statistic calculation. bioRxiv. 2016:060012.
2. Wu T, Hu E, Xu S, Chen M, Guo P, Dai Z, et al. clusterProfiler 4.0: A universal enrichment tool for interpreting omics data. Innovation (Camb). 2021;2(3):100141.
3. Ritchie ME, Phipson B, Wu D, Hu Y, Law CW, Shi W, et al. limma powers differential expression analyses for RNA-sequencing and microarray studies. Nucleic Acids Res. 2015;43(7):e47.
